# Bedside emergency cardiac ultrasound in children

**DOI:** 10.4103/0974-2700.66535

**Published:** 2010

**Authors:** Stephanie J Doniger

**Affiliations:** Department of Emergency Medicine, Children’s Hospital & Research Center, Oakland 747, 52^nd^ Street, Oakland CA 94609

**Keywords:** Bedside ultrasound, echocardiography, emergency physicians, pediatrics, pediatric emergency medicine, resuscitation

## Abstract

Bedside emergency ultrasound has rapidly developed over the past several years and has now become part of the standard of care for several applications. While it has only recently been applied to critically ill pediatric patients, several of the well-established adult indications may be applied to pediatric patients. One of the most important and life-saving applications is bedside echocardiography. While bedside emergency ultrasonography does not serve to replace formal comprehensive studies, it serves as an extension of the physical examination. It is especially useful as a rapid and effective tool in the diagnosis of pericardial effusions, tamponade and in distinguishing potentially reversible causes of pulseless electrical activity from asystole. Most recently, left ventricular function and inferior vena cava measurements have proven helpful in the assessment of undifferentiated hypotension and shock in adults and children. Future research remains to be carried out in determining the efficacy of bedside ultrasonography in pediatric-specific pathology such as congenital heart disease. This article serves as a comprehensive review of the adult literature and a review of the recent applications in the pediatric emergency department. It also highlights the techniques of bedside ultrasonography with examples of normal and pathologic images.

## INTRODUCTION

The field of Pediatric Emergency Medicine is a relatively new field of medicine developed in the 1980s.[1] Since its inception, several advancements have been made in order to improve the emergency care of children. In 2007, the Institute of Medicine Report described the deficit of pediatric-centered emergency care, calling for the need for more resources to be dedicated toward the emergency care of children.[2] The mere existence of this field of medicine embodies the idea that children are not just “small adults” but that they have a distinct pathology and need care and equipment tailored to their size and physiology.

Ultrasound technology was originally introduced in the 1950s and portable scanners made their way into the adult emergency department in the 1980s.[3,4] The first “ultrasonic stethoscope” was introduced to make rapid decisions and perform estimation of the sizes of left-sided heart structures.[5] In 1994, the first emergency ultrasound curriculum was introduced,[6] and it is now a requirement that Emergency Medicine residency programs offer training and credentialing for bedside ultrasound in their residencies. The American College of Emergency Physicians (ACEP) published 2008 recommendations for bedside ultrasound indications and guidelines for training, credentialing and developing continuing quality assurance.[7]

The use of bedside ultrasound is only beginning to emerge in the field of Pediatric Emergency Medicine and has not yet been formally incorporated into the subspecialty fellowship curricula. While numerous applications for pediatric patients are being realized, there is a strong desire for pediatric-specific training.[8] Thus far, the majority of obstacles have been identified as lack of equipment, resources, training and individuals to oversee quality assurance and credentialing.[9] Further, there are no well-established indications for children as of yet, and it has been argued that several of the indications for bedside ultrasound are different for children.[10] However, the adult applications of focused assessment sonography for trauma (FAST), first trimester pregnancy, limited echocardiography and as a procedural adjunct have been extended for use in pediatric and adolescent patients.[11] It is an especially attractive modality for children as it involves no ionizing radiation and has the potential to decrease the use of radiographs and computed tomography scans.

One such life-saving application for bedside or point-of-care ultrasonography is echocardiography. Echocardiography is the gold standard for the diagnosis of cardiac and pericardial abnormalities and is a non-invasive modality for detecting life-threatening cardiac conditions. The incorporation of bedside echocardiography has been shown to have a significant impact on medical decision-making and is a useful adjunct to the clinical exam when radiologic and laboratory studies are often unreliable and non-specific.[12,13] A recent comprehensive literature review (1978–2004) reported a remarkable increase in the diagnostic accuracy by cardiologists and non-cardiologists when a cardiac ultrasound study was added to the physical examination.[14] In the approach of the cardiac assessment in the Emergency Department, it is important to note that bedside ultrasonography is not meant to replace formal, comprehensive echocardiography. Rather, it is best seen as an extension of the physical examination and is meant to answer specific binary yes/no questions such as “Is there a pericardial effusion? Is cardiac activity present?”[15] Overall, it has been shown that non-cardiologists can be quickly and effectively trained to perform and interpret limited echocardiograms.[16] Even trainees with limited ultrasound experience have been shown to be able to successfully assess left ventricular function and pericardial effusions.[17–19]

Thus far, well-established indications for bedside emergent echocardiography in adult populations include the evaluation of blunt and penetrating chest trauma and critically ill patients. Emergency physicians may rapidly and effectively assess the presence of pericardial effusion, tamponade, cardiac activity, global assessment of contractility and the detection central volume status.[20] In patients presenting in pulseless electrical activity (PEA), the emergency physician can expeditiously differentiate cardiac standstill from correctable causes of PEA. The ACEP specifies bedside cardiac applications including the presence of cardiac activity, pericardial effusion and PEA. These assessments may be performed often, simultaneously with the physical examination, during resuscitation and as a procedural adjunct.[7,21] In those patients presenting with undifferentiated hypotension, the emergency physician can easily assess global cardiac wall function by visual interpretation and estimate central venous pressure (CVP) by measurements of the inferior vena cava (IVC).[22]

To date, there is a paucity of research dedicated toward the application of bedside echocardiography in pediatric patients. Potential extensions of adult applications to pediatric populations include evaluation for the presence of a pericardial effusion and in cardiac arrest (asystole and PEA). Additional applications may include the assessment of global wall motion and function and the assessment of undifferentiated hypotension.[23,24]

## APPLICATIONS

### Pericardial effusions, tamponade

One of the earliest and most well-recognized applications for bedside echocardiography is for the identification of pericardial effusions in traumatic and non-traumatic conditions. It is especially crucial to promptly identify pericardial effusions and tamponade as these may lead to life-saving interventions. Mandavia *et al*. studied bedside ECHO performed by emergency physicians in patients at a high risk for pericardial effusions, revealing an overall accuracy of 98%, sensitivity of 96% and specificity of 98%.[25] Pericardial effusions may arise from acute processes such as trauma or chronic effusions associated with neoplastic diseases, bacterial and fungal infections and human immunodeficiency virus.[12] On sonography, a pericardial effusion appears as an anechoic or dark stripe surrounding the heart. Early effusions appear first at the posterior pericardium and eventually develop anteriorly, ultimately creating a circumferential effusion.

In trauma patients, Ma *et al*. reported a 100% sensitivity, 99% specificity and 99% overall accuracy for the identification of pericardial effusions in patients with chest trauma.[26] In a 10-year retrospective review, Plummer *et al*. showed that bedside ECHO improved the outcome of patients with penetrating chest trauma with shorter time to diagnosis (15.5 vs. 42.4 min) and better overall survival (100% vs. 57%).[27] In a case series, Schiavone *et al*. highlighted the importance of the ability to identify pericardial effusion and early signs of tamponade in facilitating surgical management of patients with cardiac rupture secondary to blunt non-penetrating chest trauma.[28] These results contributed to the American College of Surgeons revising the Advanced Trauma Life Support (ATLS) course to incorporate the rapid bedside ultrasound assessment to identify pericardial effusions.[29,30]

Of particular clinical importance is the development of tamponade, which may lead to cardiovascular collapse and, ultimately, death. Cardiac tamponade is defined as significant compression of the heart by accumulating pericardial contents and fluid.[31] While as little as 150 mL of pericardial fluid may cause tamponade when developed acutely, a more chronic process may have a much larger volume of fluid without creating tamponade. This development of fluid accumulation leads to increased intrapericardial pressure, progressive limitation of ventricular diastolic filling and reduction of stroke volume and cardiac output.[32] Patients may present with non-specific clinical findings of pulsus paradoxus, hypotension and tachycardia. On physical examination, tamponade classically presents as Becks Triad, which consists of jugular venous distension, muffled heart tones and hypotension.[33] However, only 1/3 of the patients with tamponade will have all three features and 10% of the patients will not have any of them.

Echocardiography is the most sensitive and specific means of detecting tamponade[34] and has the potential to detect early signs of tamponade before the patient becomes unstable.[15] Features of sonographic tamponade include a circumferential pericardial effusion with a hyperdynamic heart that may exhibit right atrial compression during late diastole and right ventricular collapse during early diastole, also known as “scalloping.” Other sonographic findings may include abnormal mitral valve motion, a dilated IVC with lack of inspiratory collapse and a “swinging heart.”[32] A swinging heart is the counter-clockwise rotational movement of the heart.

### Pericardiocentesis

The treatment of tamponade includes emergent pericardiocentesis or drainage of the pericardial fluid. “Few medical situations exist in which a simple, quickly performed medical procedure can result in immediate life-saving results.”[35] The routine use of ultrasound is strongly encouraged as it is the most effective means of reducing complications.[36] Before the advent of ultrasonography for pericardiocentesis, the complication rate associated with the blind approach was approximately 7–50%. However, ultrasonography is the most effective means of reducing complications associated with pericardiocentesis. A study of ultrasound-guided pericardiocentesis in pediatric patients showed a 99% success rate, with 93% on the 1^st^ attempt and a 1% major complication and a 3% minor complication rate.[32]

### Asystole vs. PEA

Approximately 16,000 American children suffer out-of-hospital cardiac arrest per year (8–20/100,000). When compared with adults, children are more likely to survive arrest, with greater than 10% of the children surviving to hospital discharge. Among those treated for in-hospital cardiac arrest, 2/3 can be successfully resuscitated with return of spontaneous circulation and greater than 25% survive to hospital discharge.[37] Overall, cardiac arrests presenting with asystole or PEA have worse survival outcomes when compared with patients presenting with ventricular fibrillation or pulseless ventricular tachycardia. However, children have better outcomes while following asystole and PEA when compared with adults.[38]

Pulseless cardiac arrest is the cessation of cardiac activity and the absence of palpable central pulses. In children, it is especially important to determine whether a pulse is present to initiate early basic and advanced life support to improve survival.[39] Unfortunately, pulse palpation is extremely unreliable in diagnosing pediatric cardiac arrest, with an accuracy of only 78%.[40] However, ultrasonography can reliably distinguish cardiac activity from standstill and can be performed in 10 s, the time recommended to perform a pulse check. One can rapidly correlate the absence of left ventricular motion and the presence or absence of a pulse.[41] Once the physician determines that the child is pulseless, it is crucial to identify potentially correctable causes of PEA, which include hypovolemia, hypothermia, hypo or hyperkalemia, acidosis, hypoxia, tamponade, tension pneumothorax, toxins and thromboembolism.[42] Of these, bedside ultrasonography has the potential to rapidly identify tamponade, tension pneumothorax, thromboembolism and hypovolemia. It has been shown that in adult patients with PEA or near-PEA, emergency physicians can detect pericardial effusions with correctable etiologies and distinguish it from true PEA with ventricular standstill.[43] Moreover, it has been suggested that bedside ultrasonography be incorporated into the resuscitation sequence as: Airway (A), Breathing (B), Circulation (C), Defibrillation (D) and Goal-Directed ECHO (E),[44] or within each step of the ABCDE assessment.[45]

It is difficult to predict survival outcomes from pulseless cardiac arrest. Pediatric resuscitations may be for extended periods of time, often devoting considerable resources. Bedside echocardiography has the potential to give us more information that may ultimately allow us to predict survival outcomes, and terminate resuscitative efforts in futile situations. In a study of adult patients with out-of-hospital cardiac arrest, no patients who required greater than two doses of epinephrine or who had greater than 20 min of resuscitation without Return of spontaneous circulation (ROSC) survived.[46] Blaivas *et al*. and Salen *et al*. showed that no patients with sonographic cardiac standstill had ROSC, regardless of the initial presenting rhythm.[47,48] Conversely, the presence of sonographic-identified cardiac activity at any point during the resuscitation was associated with 37% survival to hospital admission. During this particular study, 96% of the physicians reported that cardiac ultrasonography provided useful information about the resuscitation and that it could be performed with ease without interfering with the resuscitation efforts.[49] It has been further suggested that low capnography in conjunction with sonographic cardiac standstill may be associated with poor resuscitation outcomes.[49,50]

### Undifferentiated shock, hypotension

A common presenting problem in the emergency department is dehydration. This is especially common in pediatrics, with approximately 9% of the children presenting diagnoses.[51] Unfortunately, the hydration status is often difficult to assess as physical examination findings and laboratory studies are often inconsistent and unreliable. As dehydration progresses, shock may develop, which is defined as the failure of the circulatory system to provide adequate oxygen delivery to the vital organs. The types of shock may be categorized as hypovolemic, distributive and cardiogenic.[34] It is often difficult to distinguish between the different etiologies of shock, but it is important that appropriate treatment be administered.

The sonographic assessment of left ventricular function or ejection fraction may be useful in patients presenting with unexplained hypotension or shock. This may expeditiously narrow the differential diagnosis (i.e., distinguishing cardiogenic from hypovolemic shock) to begin early aggressive and appropriate resuscitation.[12] Bedside ultrasound may also assist in monitoring the effectiveness of ionotropic support or vasopressor infusion and aid in the optimization of therapy.[44] Randazzo *et al*. reported that with limited focused training, emergency physicians can assess the left ventricular (LV) jection fraction accurately.[52]

Another means by which ultrasound can aid in evaluating shock and hydration status is the estimation of CVP by performing measurements of the IVC. Echocardiograph measurement of the IVC was first introduced as an estimation of right-sided cardiac function[53] and was initially studied in dialysis patients.[54] Lyon *et al*. studied the IVC diameter in relation to a 450 mL blood donation. Results suggested that IVC measurements could be a reliable indicator of even small amounts of blood loss.[55] However, because the IVC diameter correlates with body height and body surface area, it has been difficult to use the IVC measurement as a reliable and reproducible method of determining hydration status. Rather, studies have suggested using measurements in relation to the respiratory cycle and as a ratio to the diameter of the aorta.[51,56,57] During normal respiration, negative intrathoracic pressure draws blood from the highly compliant IVC to the right atrium, causing the IVC to collapse. While the absolute size varies between patients, one can grossly estimate whether the CVP is very high or very low by measuring the maximum and minimum diameters during respiration.[12] With dehydration states, the IVC will show almost complete collapse with respirations. These measurements may be used alone or in conjunction with a calculated cardiac index to non-invasively evaluate cardiac function and volume status with good correlation with CVP.[58]

In a recent study, dehydrated children were compared with normally hydrated pediatric patients presenting to the emergency department. The maximum and minimum IVC diameter measurements were lower and there was greater collapse in clinically dehydrated patients, suggesting that a collapsed IVC may reliably predict dehydration.[51] However, there are currently no clear IVC diameter reference values for pediatric patients. It has recently been suggested that because the aorta does not change with hydration status and is also correlated with body surface area (BSA) age and sex, the IVC/aorta ratio may be a reliable, easy method of assessing the hydration status.[57] Chen *et al*. showed that this ratio was lower in clinically dehydrated children and that it increased with the administration of IV fluid boluses.[56] This evaluation therefore has the potential to evaluate ongoing blood loss and monitor patient responses to fluid resuscitation.

The UHP ultrasound protocol utilized the three sonographic applications of identifying free fluid, qualitative cardiac assessment and abdominal aorta as part of the evaluation of an adult patient in PEA or with undifferentiated hypotension.[59] In pediatric patients, Pershad *et al*. presented the bedside limited echocardiography by emergency physicians, which combined a protocol of LV function estimation and IVC measurement to apply to the assessment of a pediatric patient presenting with undifferentiated hypotension or shock. When over-read by pediatric cardiologists, the emergency physicians were able to obtain acceptable quality images and accurately assess the left ventricular function and IVC volume.[60]

### Pediatric-specific pathology

The incidence of congenital heart disease in the United States is approximately 8-10 cases per 1,000 live births. The age at presentation can vary according to the specific type of lesion. Further, while cardiac emergencies are uncommon in children and the majority of children do not present with ischemic heart disease, the majority of cardiac pathology encompasses congenital heart disease. Such anatomic lesions include tetralogy of Fallot, hypoplastic left heart, atrial septal defects (ASDs) and ventricular septal defects (VSDs) to name just a few. While the identification of particular congenital heart lesions is complex and beyond the scope of bedside echocardiography, patients may present with associated pathology that immediately affects cardiac care.[24]

Of particular concern is the infant who presents with shock and it is difficult to distinguish between primary respiratory pathology and congenital heart disease associated with ductal closure. Pershad *et al*. highlighted cases of cardiac disease masquerading as acute bronchospasm. Further cardiac presentations include adolescents who may be evaluated for “sentinel events” and are at risk for sudden cardiac death. Other pathologies include infective endocarditis, hypoxemic attacks (congenital heart disease with reduced pulmonary blood flow) and acute rheumatic fever.[35] Bedside echocardiography can promptly and easily address specific questions and identify ventricular dysfunction and pericardial effusions. This may be especially important where no pediatric subspecialists are readily available.[61] There remains much research to be performed in determining the utility of bedside ultrasound to assess for congenital heart disease and to facilitate the care of the children who present with them.

## TECHNIQUE

**Figure 1:**
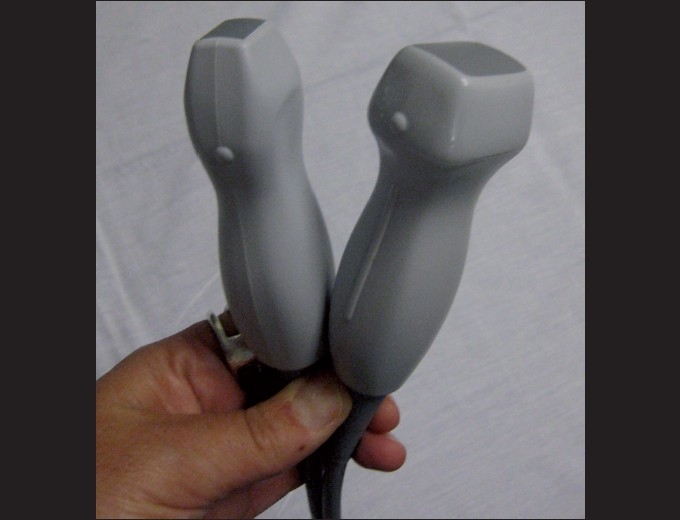
Curvilinear transducers (2-5 MHz) ideal for bedside cardiac utrasonography. Note the small footprint that can easily scan in between the rib spaces

Low-frequency transducers should be used for the cardiac assessment with a 2–5 MHz curvilinear probe with a small footprint to allow for imaging between the ribs [Figure 1]. While there is much controversy surrounding probe orientation,[62] emergency ultrasonographers often suggest using the abdominal setting on the machine to avoid confusion. It is important to note that the “cardiac” settings on machines flips the image on the screen. Therefore, in order to create a screen image that is consistent with echocardiographers, the probe marker needs to be rotated 180 degrees in relation to those transducer positions traditionally used by cardiologists.

When approaching a pediatric patient, in particular, positioning and patient comfort is paramount. Smaller children often require more preparation and patience. Measures to improve patient comfort and compliance with examinations include warmed gel, laying in the parents lap and pacifiers or feeding during the examination. Because children have much smaller and superficial structures than adult patients, it is important to adjust the depth of the images accordingly.

Standard views for bedside echocardiography include subxiphoid, parasternal long-axis, parasternal short-axis and apical 4-chamber views. Angling and tilting of the transducer may be necessary to obtain images. It is important to note that all abnormal findings should be confirmed with several views.[7,21] Additional assessments involve IVC measurements for hydration status.

### Subxiphoid

**Figure 2:**
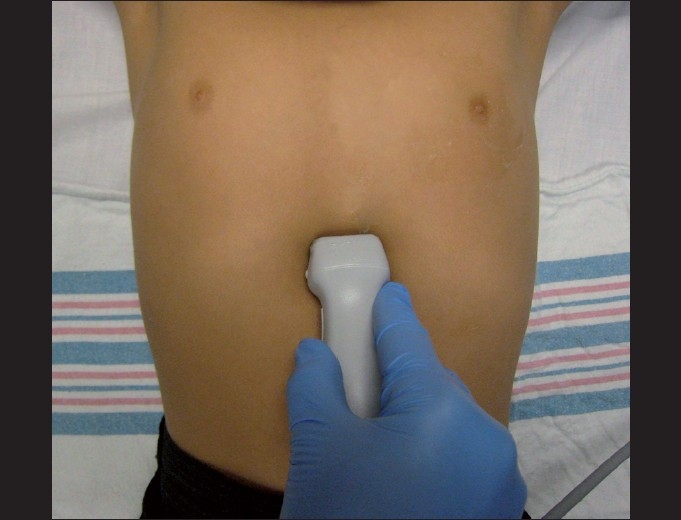
The subxiphoid view. The transducer is oriented with the probe marker toward the patient’s right and at a 15-degree angle to the patient

The subxiphoid view is the preferred view in the FAST examination and it is most useful during resuscitation as it does not typically interfere with resuscitation procedures. The probe marker should be oriented to the patient’s right side and should be placed subcostally, just below the xiphoid process, directing the probe in the direction of the patient’s left shoulder. The probe itself is quite flat and should be held at a 15-degree angle to the patient’s body, in contrast to most other positionings that are perpendicular to the patient’s body [Figure 2].

**Figure 3:**
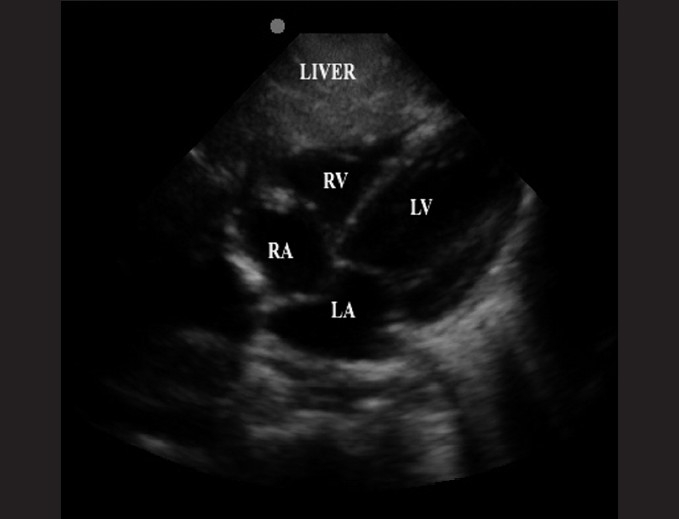
The subxiphpoid view, with the liver shown anteriorly (RV: right ventricle; LV: left ventricle; RA: right atrium; LA: left atrium)

**Figure 4:**
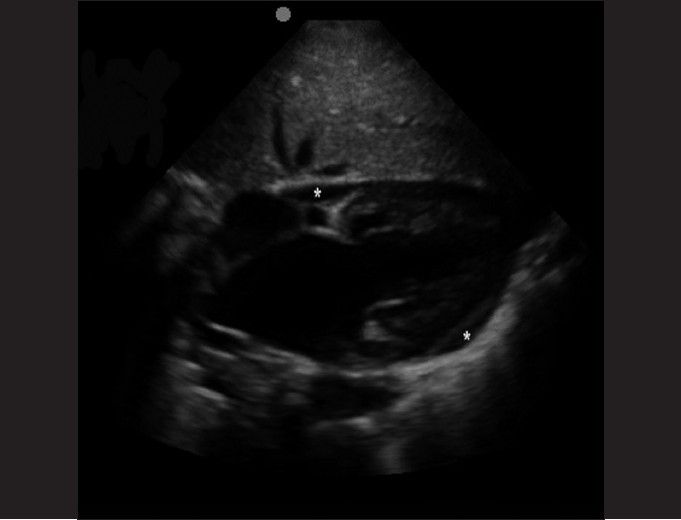
Subxiphoid view of a 2-year-old child with dilated cardiomyopathy and a small circumferential pericardial effusion

The subxiphoid view utilizes the liver as an acoustic window and readily visualizes all four chambers of the heart [Figure 3]. It is most accurate in identifying pericardial effusions, especially at the posterior pericardium where effusions begin to develop. It may be difficult to obtain in obese patients. An example of an abnormal subxiphoid view is of a child with dilated cardiomyopathy and a small circumferential pericardial effusion [Figure 4].

### Parasternal long-axis

**Figure 5:**
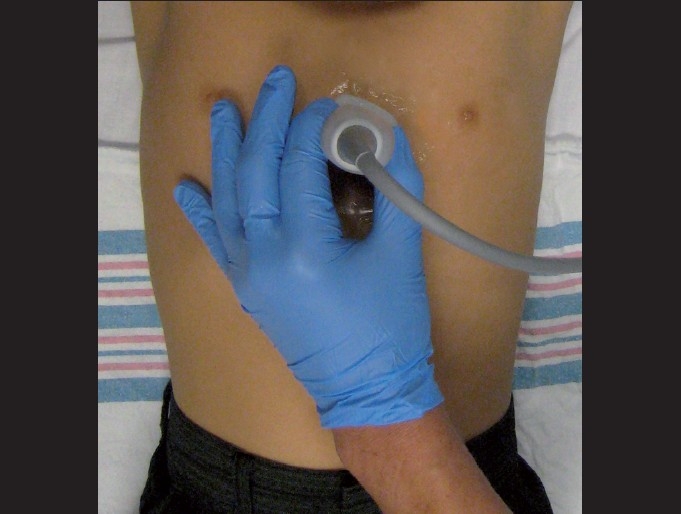
The parasternal long-axis view. The transducer is placed just lateral to the sternum and is oriented with the probe marker toward the patient’s left hip, or in the 4:00 direction, perpendicular to the patient

**Figure 6:**
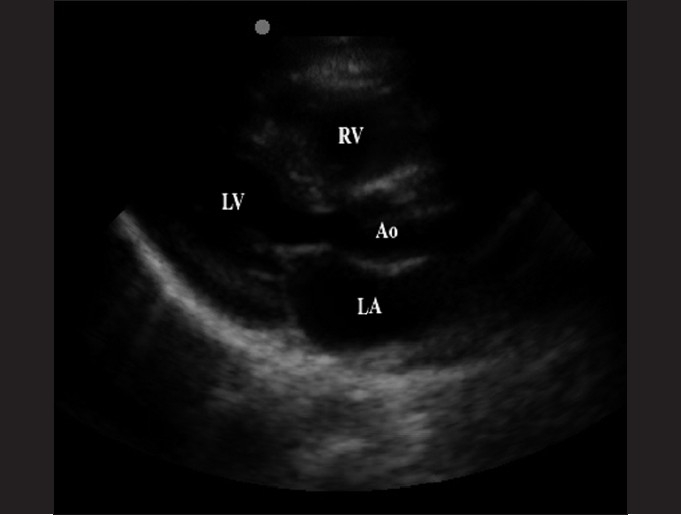
The parasternal long-axis view (LV, left ventricle; RV, right ventricle; Ao, aortic outflow tract; LA, left atrium)

The parasternal long-axis view is the preferred view for the assessment of left ventricular contractility and for the identification of pericardial effusions when adequate subxiphoid images cannot be obtained. The probe is placed perpendicular to the chest wall, immediately to the left of the sternum, between the 3^rd^ and 4^th^ intercostal space, above the level of the nipple line. The probe marker should be directed toward the patient’s left hip or in 4:00 direction [Figure 5]. A distinguishing feature is that the aortic outflow tract (Ao) is stacked on top of the left atrium [Figure 6].

The parasternal long-axis view is best utilized to measure contractility. In the review of methods of sonographically determining LV function, McGowan *et al*. reported that visual estimation of cardiac function into normal mild, moderate or severe dysfunction is as good or better than other more complex, calculated methods.[63] The left ventricular shortening fraction, which is expressed as a percentage, may be calculated by the formula: (end diastolic dimension – end systolic dimension)/end diastolic dimension. A normal calculated shortening fraction is greater than 30%.[60]

### Parasternal short-axis

**Figure 7:**
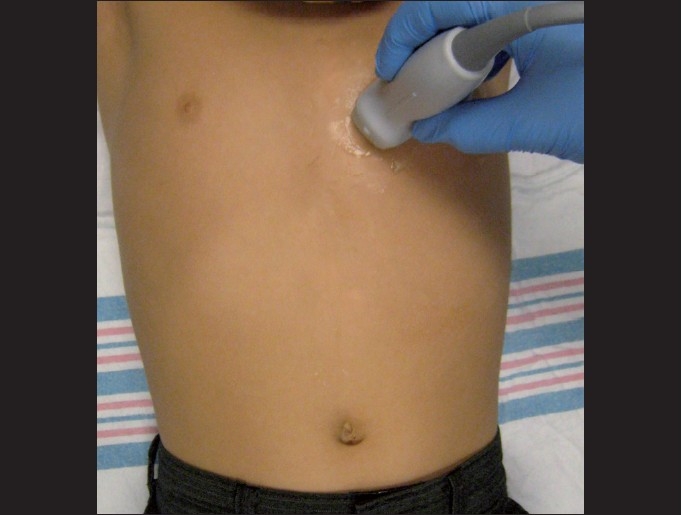
The parasternal short-axis view. The transducer is oriented with the probe marker toward the patient’s right hip, or in the 7:00 direction. This is a 90-degree rotation from the parasternal long-axis view

**Figure 8:**
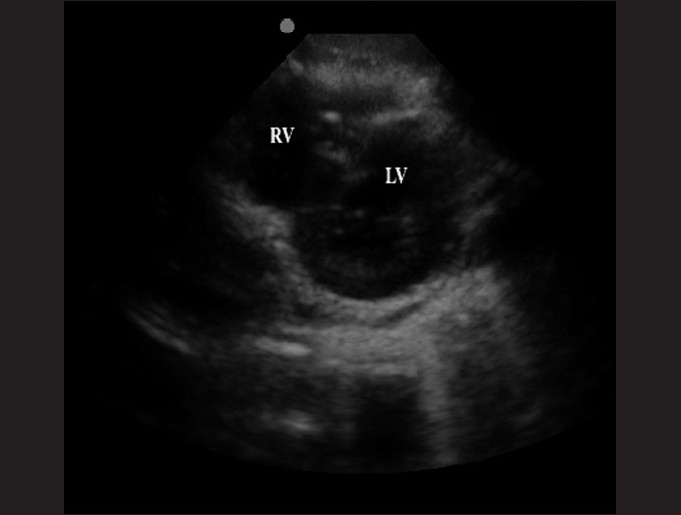
The parasternal short-axis view, also known as the “doughnut” or “fish-mouth” view, of the left ventricle (LV)

The parasternal short-axis view readily evaluates the contractility and valvular function. Once the parasternal long-axis view is obtained, the transducer should be rotated 90 degrees in order to obtain the parasternal short-axis view. The probe marker should be directed toward the patient’s right hip or in the 6:00 direction [Figure 7]. One can sweep the transducer from the base of the heart to the apex to visualize various levels of the heart in cross-section from the level of the mitral valve, papillary muscles and tricuspid valve. The distinguishing feature of the parasternal short-axis view is that the left ventricle is circular with a valve in the middle, resembling a “fish-mouth” or “doughnut” [Figure 8].

### Apical 4-chamber

**Figure 9:**
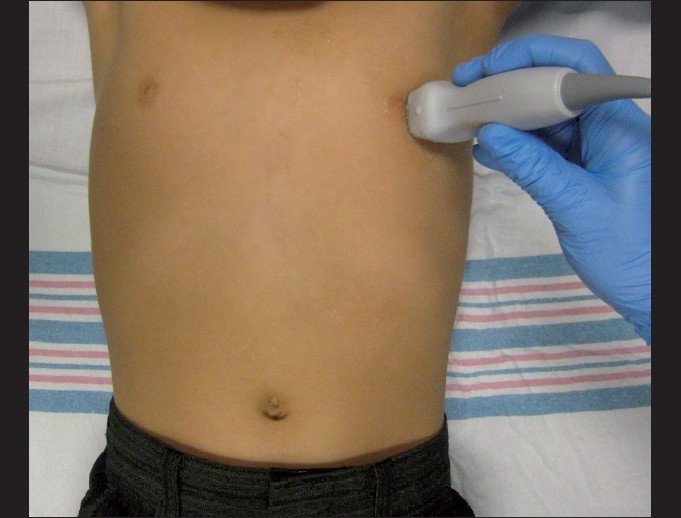
The apical 4-chamber view. The transducer is placed over the point of maximal impulse, oriented with the probe marker toward the patient’s right

**Figure 10:**
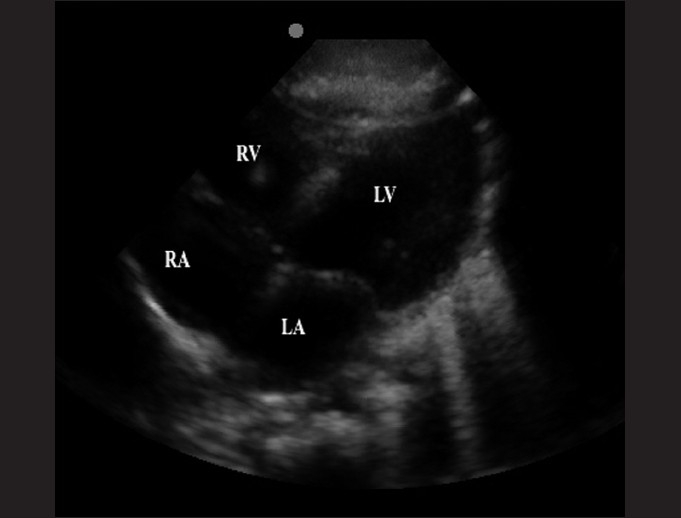
The apical 4-chamber view (RV: right ventricle; LV: left ventricle; RA: right atrium; LA: left atrium)

**Figure 11:**
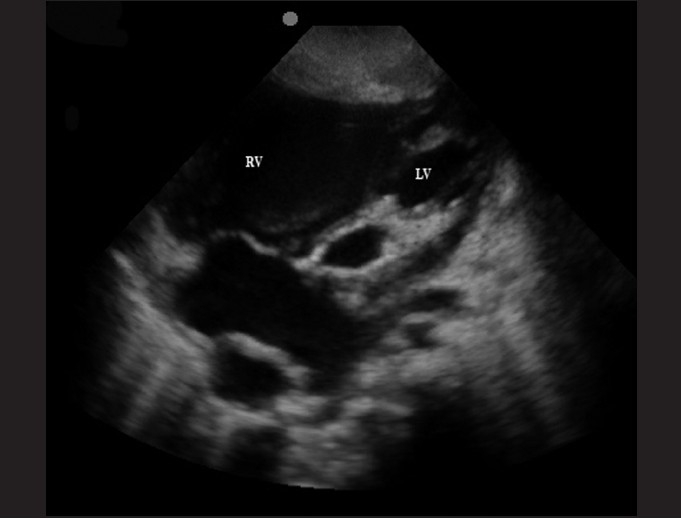
The apical 4-chamber view of a 5-year-old child with repaired Hypoplastic Left Heart Syndrome. Note the extremely small size of the left ventricle (LV) in relation to the right ventricle (RV)

The apical 4-chamber view is the preferred view for evaluating the relative dimensions of the right and left sides of the heart. The transducer is placed at the cardiac apex, which may be located by palpating the point of maximal impulse (PMI). This is typically located at the T4-T5 level, at the 5^th^ intercostal space, just lateral to the nipple. The probe marker is typically oriented toward the patient’s right side [Figure 9]. The patient often needs to be rotated to his left side in order to bring the heart more anterior to the chest wall and to decrease artifact associated with the left lung [Figure 10]. An example of an abnormal apical 4-chamber is a child with repaired Hypoplastic Left Heart Syndrome [Figure 11].

### IVC

**Figure 12:**
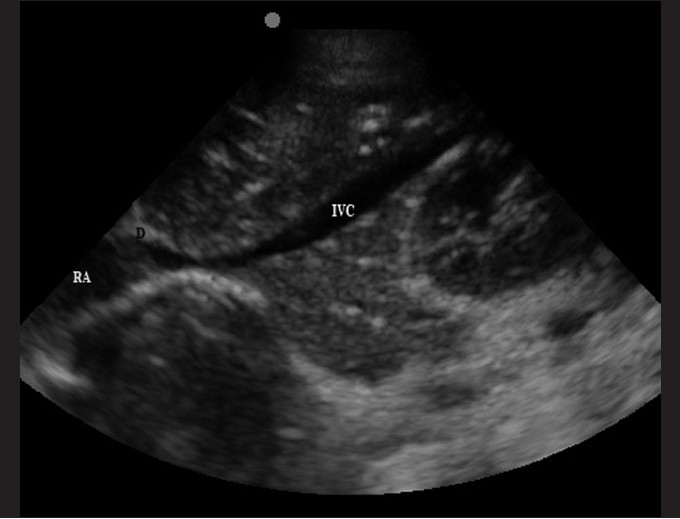
The sagittal or longitudinal view of the IVC (RA: right atrium; D: diaphragm; IVC: inferior vena cava)

**Figure 13:**
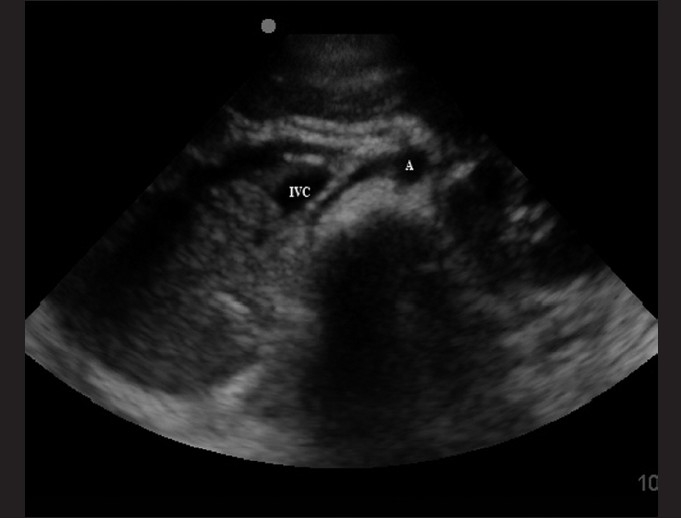
Transverse or cross-sectional view of the inferior vena cava (IVC) in relation to the aorta (A)

The transducer is placed in the subxiphoid position [Figure 3] and the IVC can be traced as it travels behind the liver.[15] A normal IVC size extrapolated from adult data is 9.2 ± 2.4 mm/m^2^, which collapses by 50% during quiet inspiration.[60] Absolute measurements of the IVC have not been shown to be as effective as percent of collapse during respiratory variation.[58] In adult patients, this measurement has been made within 3–4 cm of the entrance into the right atrium in the longitudinal view or 2 cm distal to the hepatic vein confluence. At this location in the sagittal view, the anterior and posterior walls are parallel [Figure 12]. One can measure the maximal and minimal diameters of the IVC with respiration. This is best obtained in the M-mode, with a cine-loop to evaluate several respiratory cycles. An additional method is to compare the ratio of IVC to aorta diameters. The aorta lies adjacent to the IVC (to the right), just anterior to the vertebral body [Figure 13]. A proposed IVC/aorta index value has been suggested as 1.2 ± 2 SD (for SD = 0.17).[58]

### M-mode: Asystole

**Figure 14:**
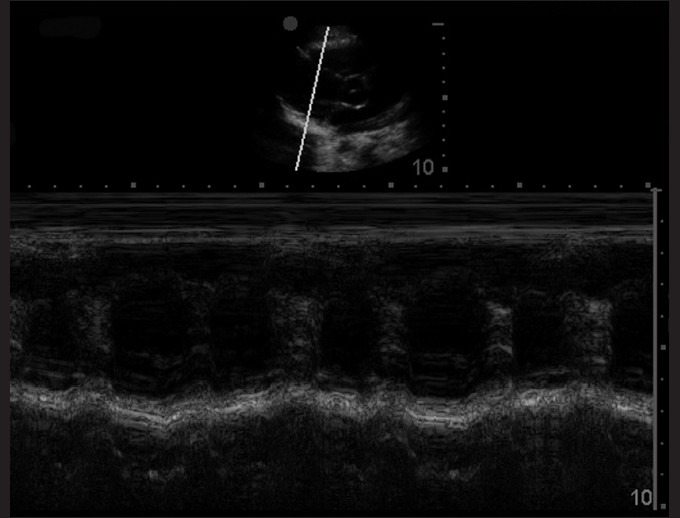
M-mode of normal cardiac activity. When the cursor is placed over the left ventricular walls, cardiac contractions are plotted on the one-dimensional representation of motion

**Figure 15:**
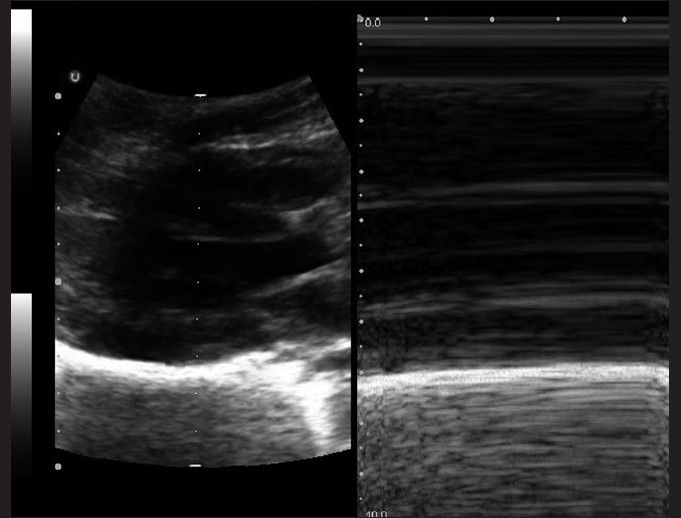
M-mode of asystole with absence of cardiac contractions

M-mode, or motion mode, allows for a one-dimensional tracing of structure movement over time. In the parasternal long-axis view, the cursor may be placed over the walls of the left ventricle in order to assess for contractility [Figure 14]. This is the most effective method for evaluating asystole [Figure 15]. It is important to note that while performing this assessment to assess for cardiac contractions, the compressions and artificial respirations must be held in order to avoid motion from external sources other than from the ventricle.

### Pericardiocentesis

**Figure 16:**
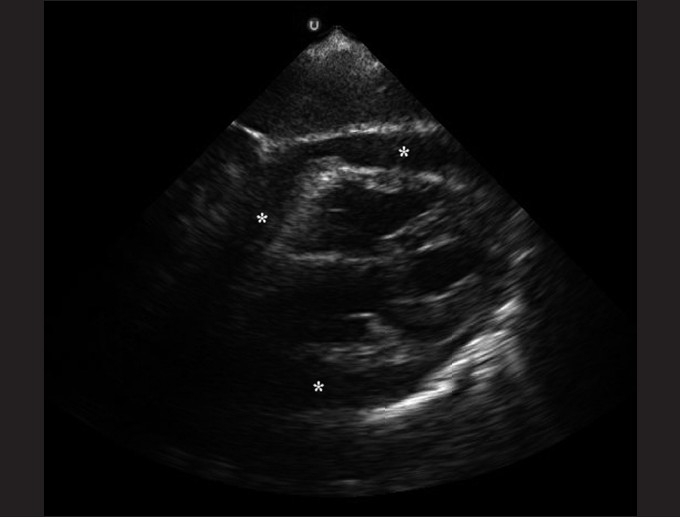
Subxiphoid view of pericardial tamponade with a large circumferential pericardial effusion and right ventricular scalloping

Tamponade is the indication for pericardiocentesis [Figure 16]. The parasternal long-axis approach provides a more direct anatomic approach and direct visualization of the needle. This approach is associated with less cardiac lacerations, pneumothoraces, pneumoperitoneum and liver lacerations associated with pericardiocentesis.[64]

## CONCLUSIONS

While bedside ultrasound has only recently been incorporated into the practice of Pediatric Emergency Medicine, several well-established adult applications may be applied toward the care of pediatric patients. Most notable are the identification of pericardial effusions and tamponade, the assessment of PEA and asystole, global contractility and IVC evaluation for hydration status. More research remains to be performed with the application of bedside ultrasonography with regards to the early recognition of congenital heart disease and for other pediatric-specific pathologies.
